# Activation of ChTLR15/ChNF-κB-ChNLRP3/ChIL-1β signaling transduction pathway mediated inflammatory responses to *E. tenella* infection

**DOI:** 10.1186/s13567-020-00885-8

**Published:** 2021-01-29

**Authors:** Jian Li, Xuelian Yang, Zhipeng Jia, Chunli Ma, Xinghui Pan, Dexing Ma

**Affiliations:** 1 College of Veterinary Medicine, Northeast Agricultural University, Harbin, 150030 Heilongjiang China; 2 Heilongjiang Key Laboratory for Experimental Animals and Comparative Medicine, Harbin, 150030 Heilongjiang China; 3 Shandong Vocational Animal Science and Veterinary College, Weifang, 261061 Shandong China; 4 College of Food Science, Northeast Agricultural University, Harbin, 150030 Heilongjiang China

**Keywords:** *E. tenella* sporozoites, ChTLR15, ChNLRP3, Inflammatory responses

## Abstract

Avian coccidiosis caused by *Eimeria* leads to severe economic losses in the global poultry industry. Although chicken Toll-like receptor 15 (ChTLR15) was reported to be involved in *Eimeria* infection, the detailed mechanism underlying its role in the inflammatory response remains to be discovered. The present study demonstrated that the mRNA expression levels of ChTLR15, ChMyD88, ChNF-*κ*B, ChNLRP3, ChCaspase-1, ChIL-18 and ChIL-1β and the protein levels of ChTLR15 and ChNLRP3 in cecal tissues of *Eimeria*-infected chickens were significantly elevated at 4, 12, and 24 h compared with those in noninfected control chickens (*p* < 0.01). Moreover, the mRNA levels of molecules in the ChTLR15/ChNF-κB and ChNLRP3/ChIL-1β pathways and the protein levels of ChTLR15 and ChNLRP3 in chicken embryo fibroblast cells (DF-1) stimulated by *E. tenella* sporozoites were consistent with those in *Eimeria*-infected chickens. Furthermore, overexpression of ChTLR15 in DF1 cells augmented activation of the ChTLR15/ChNF-κB and ChNLRP3/ChIL-1β pathways when stimulated with *E. tenella* sporozoites, while knockdown of ChTLR15 in DF1 cells showed inverse effects. Taken together, the present study provides evidence that *E. tenella* sporozoites specifically activate ChTLR15 and then trigger activation of the ChNLRP3/ChIL-1β pathway, which partially mediates inflammatory responses to *Eimeria* infection.

## Introduction

Avian coccidiosis caused by the intracellular protozoan *Eimeria* is responsible for severe economic losses in the global poultry industry [1, 2]. *Eimeria tenella* is one of the most pathogenic species because it invades chicken cecum epithelial cells and causes severe pathological lesions in the ceca. To date, the methods for preventing and controlling coccidiosis still rely on conventional prophylactic chemotherapy and attenuated parasite vaccines. However, long-term measures against coccidiosis have gradually shown unavoidable drawbacks, including residues of chemical drugs in poultry meat, emergence of *Eimeria* strains with drug resistance, and pathogenic reversion of live parasite vaccines [3, 4]. Therefore, in recent years, researchers have gradually focused on the study of novel anticoccidial vaccines or drugs that are safer, less expensive, and more effective. A deep understanding of the mechanism of parasite invasion into host cells is a prerequisite for developing novel vaccines or drugs. Although the invasion mechanism of *Eimeria* parasites is not clear, the activation of related signal transduction pathways during *Eimeria* invasion into host cells plays a vital role [5–7]. The identification of key signaling pathways involved in *Eimeria* infection and the exploration of target inhibitors of signaling pathways may be a promising method to develop novel strategies for controlling coccidiosis.

Innate immunity is the first line of defense against invading pathogens, and therefore, exploration of signaling pathways involved in innate immunity is meaningful for understanding inflammatory responses to *Eimeria* infection and for finding novel drugs against coccidiosis. Toll-like receptors (TLRs) are pattern recognition receptors (PRRs) located on the cell surface that are responsible for recognizing conserved components of pathogens that are typically called pathogen-associated molecular patterns (PAMPs). Chicken TLR15 (ChTLR15) plays a crucial role during intestinal pathogen infection [6, 8], but ChTLR15 is not categorized as one of the known TLRs, and its specific ligand remains elusive [9]. The roles of ChTLR15 in intestinal injury caused by *Eimeria* has not been reported to date. The NOD-like receptor 3 (NLRP3) inflammasome, assembled by the sensor protein NLRP3, adaptor protein ASC and Caspase, plays a critical role in mediating inflammatory responses. Recent studies have revealed that NLRP3 activation is closely related to pathogenic bacteria and that the NLRP3/Caspase-1/IL-1β signaling pathway responds to several types of agonists and stimuli [10]. Recently, it was reported that NLRP3 plays an important role during infection of intracellular parasites, including *Trypanosoma cruzi, Toxoplasma gondii, Plasmodium spp., Leishmania spp.* and *Neospora caninum* [11–14]. However, the activation mechanism of chicken NLRP3 (ChNLRP3) and its role in the immune response to *Eimeria* infection have not been reported until now.

Our preliminary results demonstrated that the mRNA expression levels of molecules involved in the ChTLR15/ChNF-κB and ChNLRP3/ChIL-1β pathways in the cecal tissues of *Eimeria*-infected chickens were significantly elevated compared with those in the cecal tissues of noninfected control chickens (*p* < 0.01). It is generally accepted that the developmental stages of *Eimeria* in the chicken intestinal tract within two days are mainly sporozoites that invade intestinal epithelial cells and initiate subsequent development. Based on our preliminary results, we speculated that the ChTLR15/ChNF-κB-ChNLRP3/ChIL-1β signaling pathway may be specifically activated by *Eimeria* sporozoites, which mediate inflammatory injury during *Eimeria* infection. It was reported that chicken embryo fibroblast cells (DF-1) can be directly invaded by *Eimeria* sporozoites and have been extensively applied to study innate immune responses [15–17]. To verify the above hypothesis, the ChTLR15/ChNF-κB and ChNLRP3/ChIL-1β signaling pathways were analyzed in DF1 cells at the transcriptional and protein levels using small interfering RNA (siRNA) and overexpression technology. This study provides references for developing novel strategies against coccidiosis based on inhibitors of the ChTLR15/ChNF-κB-ChNLRP3/ChIL-1β signaling pathway.

## Materials and methods

### Cells and parasites

The chicken embryo fibroblast cell line DF-1 was purchased from ATCC (CRL-12203) and stored in our laboratory. The *E. tenella* strain used in the present study was originally isolated and stored in our laboratory, and it was used in our previous report [18]. *E. tenella* oocysts were propagated in 3-week-old specific pathogen-free (SPF) chickens. Sporulated oocysts were preserved at 4 ℃ in 2.5% (w/v) potassium dichromate until use.

### Preparation of polyclonal antisera against ChNLRP3 and ChTLR15 proteins

Total RNA was extracted from cecal tissues using TRIzol reagent (Invitrogen, USA) according to the manufacturer’s protocol. An objective ChNLRP3 gene fragment of 600 bp was amplified by the primer pair ChNLRP3/600-F1 and ChNLRP3/600-R1 (Table 1) using prepared cDNA as template. The amplified ChNLRP3 fragment was cloned into the pET-30a vector (Novagen, Madison, WI) to produce the plasmid pET-30a-ChNLRP3. The above plasmid was transformed into *E. coli* BL21 competent cells to generate recombinant-positive *E. coli* cells. When the optical density at 600 nm (OD600) reached 0.5, the cultured positive bacteria were induced by 1.0 mM isopropyl-b-d-thiogalactopyranoside (IPTG) for 3 h at 37 °C. A polypeptide chain of ChTLR15 (5ʹ-KTTNEPLVRAENGPN-3ʹ) synthesized by GenScript (Nanjing) Co., Ltd. was used as an immunogen to produce polyclonal antisera against the ChTLR15 protein. The expression and purification of ChNLRP3 protein and production of polyclonal antisera against ChNLRP3 and ChTLR15 protein were carried out according to previously reported methods [19].

**Table 1:** **Primer sequences with their corresponding PCR product size.**

Genes	Primers sequences (5′-3′)	Restriction recognition sites	PCR product (base pairs)
ChNLRP3	ChNLRP3/600-F1 CGGGGTACCATGGCAGGAGAAGAAAGCACCAT	Kpn I	600 bp
	ChNLRP3/600-R1 CCGCTCGAGGTCAAACTGTGTGCAGAGGGTCC	Xho I	
ChTLR15	ChTLR15/CDS-F1 ATAGAATTCATGAGGATCCTTATTGGG	EcoR I	2607 bp
	ChTLR15/CDS-R1 CCGCTCGAGTCATTCCATCTCAATTACA	Xho I	
ChTLR15	ChTLR15-F GGCTGTGGTATGTGAGAATG	–	113 bp
	ChTLR15-R ATCGTGCTCGCTGTATGA	–	
ChMyD88	ChMyD88-F CTGGCATCTTCTGAGTAGT	–	76 bp
	ChMyD88-R TTCCTTATAGTTCTGGCTTCT	–	
ChNF-κB	ChNF-κB –F TCTGAACAGCAAGTCATCCATAACG	–	253 bp
	ChNF-κB –R AAGGAAGTGAGGTTGAGGAGTCG	–	
ChNLRP3	ChNLRP3-F GGTTTACCAGGGGAAATGAGG	–	252 bp
	ChNLRP3-R TTGTGCTTCCAGATGCCGT	–	
ChCaspase-1	ChCaspase-1-F TAAGCACTTGAGACAGCGGGACG	–	245 bp
	ChCaspase-1-R GGATGTCCGTGGTCCCATTACTC	–	
ChIL-1β	ChIL-1β-F TTCCGCTACACCCGCTCACAGT	–	242 bp
	ChIL-1β-R CCGCTCATCACACACGACAT	–	
ChIL-18	ChIL-18-F CAGCGTCCAGGTAGAAGATAAG	–	210 bp
	ChIL-18-R TCCTCAAAGGCCAAGAACAT	–	
β-actin	β-actin-F GCCAACAGAGAGAAGATGACAC	–	139 bp
	β-actin-R GTAACACCATCACCAGAGTCCA	–	

The restriction enzyme sites in primer sequences are underlined.

### Detection of prepared antisera

The titers of the prepared polyclonal antisera were tested using indirect enzyme-linked immunosorbent assay (ELISA) methods as described in a previous report [20]. Western blot was used to detect the two prepared polyclonal antisera as described by Wang et al. [21]. Briefly, proteins extracted from chicken cecal tissues using RIPA Lysis Buffer (Beyotime Biotechnology) were separated by sodium dodecyl sulfate-polyacrylamide gel electrophoresis (SDS-PAGE) and then transferred to nitrocellulose membranes. The membranes were probed with prepared rabbit anti-ChNLRP3 and anti-ChTLR15 polyclonal antisera. The membranes were washed twice with TTBS (0.1% Tween 20, 50 mmol/L Tris–HCl, 150 mmol/l NaCl, pH 7.5) and reacted with horseradish peroxidase (HRP)-conjugated goat anti-rabbit IgG antibody (Sigma, USA). After washing, the target immunoreactive bands were visualized by an ECL chemiluminescence detection kit (Sangon Biotech Co., Ltd, Shanghai, China) according to the manufacturer's instructions.

### Expression levels of molecules in the ChTLR15 and ChNLRP3 pathways in chicken ceca

One-day-old white leghorn specific pathogen-free (SPF) chickens purchased from Harbin Veterinary Research Institute were randomly divided into three groups of 20 chickens each. Chickens were housed in individual cages and fed feed free of anticoccidials. At 21 days of age, each chicken in group 1 (low dosage infection group) was orally infected with 5000 *E. tenella* sporulated oocysts (500 μL), group 2 (high dosage infection group) with 30 000 *E. tenella* sporulated oocysts (500 μL), and group 3 (control group) with 500 μL of PBS (pH 7.2). Five chickens were randomly selected from each group at 4, 12, 24 and 72 h post-infection, and the ceca were sampled for quantification of the mRNA expression levels of ChTLR15, ChMyD88, ChChNF-κB, ChNLRP3, ChCaspase-1, ChIL-18 and ChIL-1β by quantitative real-time PCR (qPCR) and quantification of the protein expression levels of ChTLR15, ChNLRP3 and ChIL-1β by Western blot. Briefly, total RNA was extracted from the ceca using a GenElute™ Total RNA Purification Kit (Sigma-Aldrich). cDNA was synthesized from 1 μg of total RNA using the Prime Script RT Reagent Kit. qPCR was carried out using SYBR® Premix Ex Taq™ II (Tli RNase H Plus) (TaKaRa Biotech Corp., Dalian, China) according to the manufacturer’s instructions. qPCR was performed following the minimum information for publication of quantitative real-time PCR experiments (MIQE) guidelines [22]. The mRNA expression levels of chicken β-actin in cecal tissues proved stable in our preliminary experiment; therefore, β-actin was used as a reference gene for normalization. The primer pairs used in the present study are listed in Table 1. For each 100-fold diluted cDNA sample, the amplification efficiencies of all target genes and reference genes were similar, and the 2^−ΔΔCt^ method was used to analyze the relative quantification of target genes [23]. Cecal tissues (2 g) were homogenized in 2 mL of normal saline using a high-speed tissue homogenizer (Kinematica, Switzerland). Protein expression levels of ChTLR15 and ChNLRP3 in cecal homogenates were detected by Western blot as described by Wang et al. [21]. The protein expression levels of ChIL-1β in cecal homogenates were determined using an ELISA kit (Jiancheng Bioengineering Institute, Nanjing, China) according to the manufacturer's instructions. Animal experiments were performed based on the regulations (SRM-12) of the Ethics Committee for Animal Sciences at Northeast Agricultural University, Heilongjiang Province, PR China.

### Sporozoite purification and stimulation of DF1 cells

Sporozoites were purified from *E. tenella* sporulated oocysts according to the methods described in a previous report [18] and then adjusted to a concentration of 4 × 10^6^/mL. Chicken DF1 cells were seeded in a 6-well plate at 1.5 × 10^6^ cells per well and cultured in DMEM (Sigma) with 10% FBS (Gibco) at 41 °C. Then, monolayer DF1 cells were stimulated with 4 × 10^5^ sporozoites per well.

### Expression levels of molecules in the ChTLR15 and ChNLRP3 pathways in DF1 cells

To clarify the expression levels of molecules in the ChTLR15/ChNF-κB-ChNLRP3/ChIL-1β signaling pathway, DF1 cells were stimulated with sporozoites. At 0, 2, 4, 6, and 8 h post-stimulation, cells in each group were collected to quantify the mRNA levels of ChTLR15, ChMyD88, ChNF-κB, ChNLRP3, ChCaspase-1, ChIL-18 and ChIL-1β using qPCR. Total RNA was extracted from cells using a GenElute™ Total RNA Purification Kit (Sigma-Aldrich) according to the manufacturer’s instructions. cDNA preparation and qPCR were performed using the Prime Script RT Reagent kit and SYBR® Premix Ex Taq™ II (Tli RNase H Plus) (TaKaRa Biotech Corp., Dalian, China), respectively. The primer pairs used for qPCR are listed in Table 1. Chicken β-actin was used as a reference gene. Data were analyzed by the 2^−ΔΔCt^ method [23]. At 0, 2, 4, 6, and 8 h post-stimulation, the protein expression levels of ChTLR15 and ChNLRP3 in DF1 cells were detected by Western blot as described by Wang et al. [21], and the protein expression levels of ChIL-1β in the culture supernatant were determined using an ELISA kit (Jiancheng Bioengineering Institute, Nanjing, China).

### Interference of ChTLR15 in DF1 cells

To investigate the relationship between ChTLR15/ChNF-κB and the ChNLRP3/ChIL-1β pathway, interference with ChTLR15 was performed based on the small interfering RNA (siRNA) technique. Three siRNAs for ChTLR15 (siChTLR15#1, siChTLR15#2 and siChTLR15#3) were designed and synthesized by GenScript (Nanjing) Co., Ltd. (Table 2). To ensure the transfection efficiency of the three target siRNAs, the concentrations of terminal fluorescently labeled siRNA (FAM Negative Control) and Lipofectamine 2000 (Thermo Fisher Scientific) were optimized. Briefly, 4 μL, 5 μL and 6 μL of Lipofectamine 2000 (Thermo Fisher Scientific) were added to each well (1.5 × 10^6^ cells per well) in 6-well plates. Then, final concentrations of 40 nM, 50 nM and 60 nM FAM negative control were added. The transfection effects were observed by fluorescence microscopy.

**Table 2:** **Sequences of siRNA for ChTLR15.**

Name of siRNA	Sequences (5′-3′)
ChTLR15#1	CGACAUGCGAUAUUAGUAATT UUACUAAUAUCGCAUGUCGTT
ChTLR15#2	GAAGUUAUCUAAAUUAGAATT UUCUAAUUUAGAUAACUUCTT
ChTLR15#3	GAGCGUUCUGACUGUUCAATT UUGAACAGUCAGAACGCUCTT

To evaluate the interference effects of siChTLR15RNA#1, siChTLR15RNA#2 and siRNAChTLR15#3, three target siRNAs and negative control (NC) siRNA were transfected into DF1 monolayer cells (1.5 × 10^6^ per well) according to the optimized conditions. At 24 h post-transfection, DF1 cells in each well were stimulated with 4 × 10^5^ sporozoites for 2 h. Cells transfected with negative control (NC) siRNA and stimulated with (NC + Stim) or without (NC + Unstim) sporozoites were used as controls. The interference efficiency of the three target siRNAs was determined by quantifying the mRNA expression levels of ChTLR15, and the target siRNA showing the highest interference efficiency was chosen for subsequent experiments.

### Expression levels of molecules in the ChNLRP3 pathway in ChTLR15-knockdown DF1 cells

Monolayer DF1 cells (1.5 × 10^6^ cells per well) in a 6-well plate were treated with the selected ChTLR15 siRNA for 2 h and then stimulated with 4 × 10^5^ purified *E. tenella* sporozoites per well. At 0, 2, 4, 6, and 8 h post-stimulation, cells in each group were collected to quantify the mRNA expression levels of ChTLR15, ChMyD88, ChNF-κB, ChNLRP3, ChCaspase-1, ChIL-18 and ChIL-1β by qPCR as previously described. Protein expression levels of ChTLR15 and ChNLRP3 in cells were detected by Western blot as described by Wang et al. [21]. ChIL-1β protein expression levels in culture supernatant were determined using an ELISA kit (Jiancheng Bioengineering Institute, Nanjing, China). Cells transfected with negative control (NC) siRNA and stimulated with (NC + Stim) or without (NC + Unstim) sporozoites were used as controls.

### Overexpression of ChTLR15 in DF1 cells

The gene fragment encoding the ChTLR15 protein was amplified by the primer pair ChTLR15/CDS-F1 and ChTLR15/CDS-R1 (Table 1) using cDNA prepared from cecal tissues as a template. The amplified fragment was cloned into the pCMV vector (Novagen, Madison, WI) to construct the plasmid pCMV-ChTLR15. The above positive plasmid (4 μg) was transfected into DF1 monolayer cells (1.5 × 10^6^ cells per well) cultured in a 6-well plate using Lipofectamine 2000 (Thermo Fisher Scientific) according to the manufacturer’s protocol. At 0, 12, 24, and 36 h post-transfection, the expression levels of ChTLR15 protein were detected by Western blot as previously described to determine the optimal time for overexpressing ChTLR15 protein in DF1 cells.

### Expression levels of molecules in the ChNLRP3 pathway in ChTLR15-overexpressing DF1 cells

At 36 h post-transfection with pCMV-ChTLR15, DF1 monolayer cells (1.5 × 10^6^ cells per well) in each group were stimulated by 4 × 10^5^ sporozoites per well. Cells transfected with the empty plasmid pCMV and stimulated with or without sporozoites were used as controls. At 0, 2, 4, 6, and 8 h post-stimulation with sporozoites, cells in each group were collected to quantify mRNA levels of ChTLR15, ChMyD88, ChNF-κB, ChNLRP3, ChCaspase-1, ChIL-18 and ChIL-1β by qPCR, and data were analyzed as previously described [23]. Protein expression levels of ChTLR15 and ChNLRP3 in cells were determined by Western blot as described by Wang et al. [21]. The supernatant medium from each group was collected for detection of ChIL-1β protein using an ELISA kit (Jiancheng Bioengineering Institute, Nanjing, China). Cells transfected with empty plasmid pCMV and stimulated with (pCMV + Stim) or without (pCMV + Unstim) sporozoites were used as controls.

### Statistics analysis

The grey values of Western blot images were quantified by ImageJ software. Data are expressed as the means ± standard deviation (SD) and subjected to one-way analysis of variance (ANOVA). ANOVA with Tukey’s multiple-comparison procedures in GraphPad Prism 5 software [24] was used to compare differences between mean values. The results were considered significant at *p* < 0.05 and highly significant at *p* < 0.01.

## Results

### Detection of polyclonal antisera against ChNLRP3 and ChTLR15

**Figure 1:**
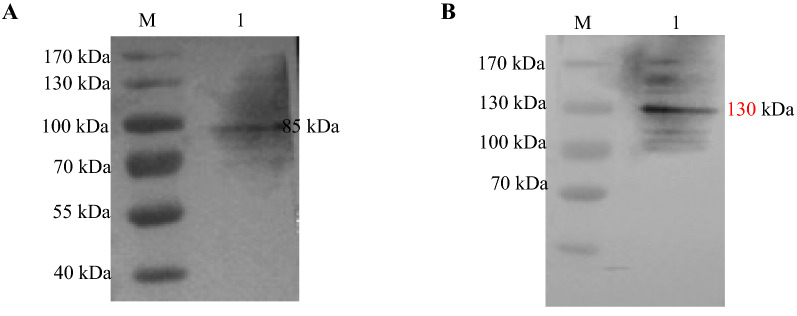
**Characterization of prepared polyclonal antisera by Western blot.** Proteins extracted from chicken cecal tissues were separated by SDS-PAGE, transferred to nitrocellulose membranes, and then reacted with the prepared rabbit polyclonal antisera against ChNLRP3 and ChTLR15. Membranes were washed and then probed with goat anti-rabbit HRP-conjugated IgG antibody (Sigma, USA). The bands for ChNLRP3 and ChTLR15 proteins were observed. **A** Lane 1, ChNLRP3 protein (85 kDa). M, protein molecular weight marker (Fermentas). **B** Lane 1, ChTLR15 protein (130 kDa). M, protein molecular weight marker (Fermentas).

The polypeptide chain of ChTLR15 and the purified recombinant ChNLRP3 protein were used to immunize rabbits. The titers of the prepared polyclonal antisera against ChNLRP3 and ChTLR15 were both 1:2^17^. Protein bands of approximately 85 kDa and 130 kDa were observed corresponding to the molecular weights of the ChNLRP3 and ChTLR15 proteins, respectively (Figure 1), which shows that the two prepared polyclonal antisera specifically react with the target protein.

### Expression levels of molecules in the ChTLR15 and ChNLRP3 pathways in the ceca

**Figure 2:**
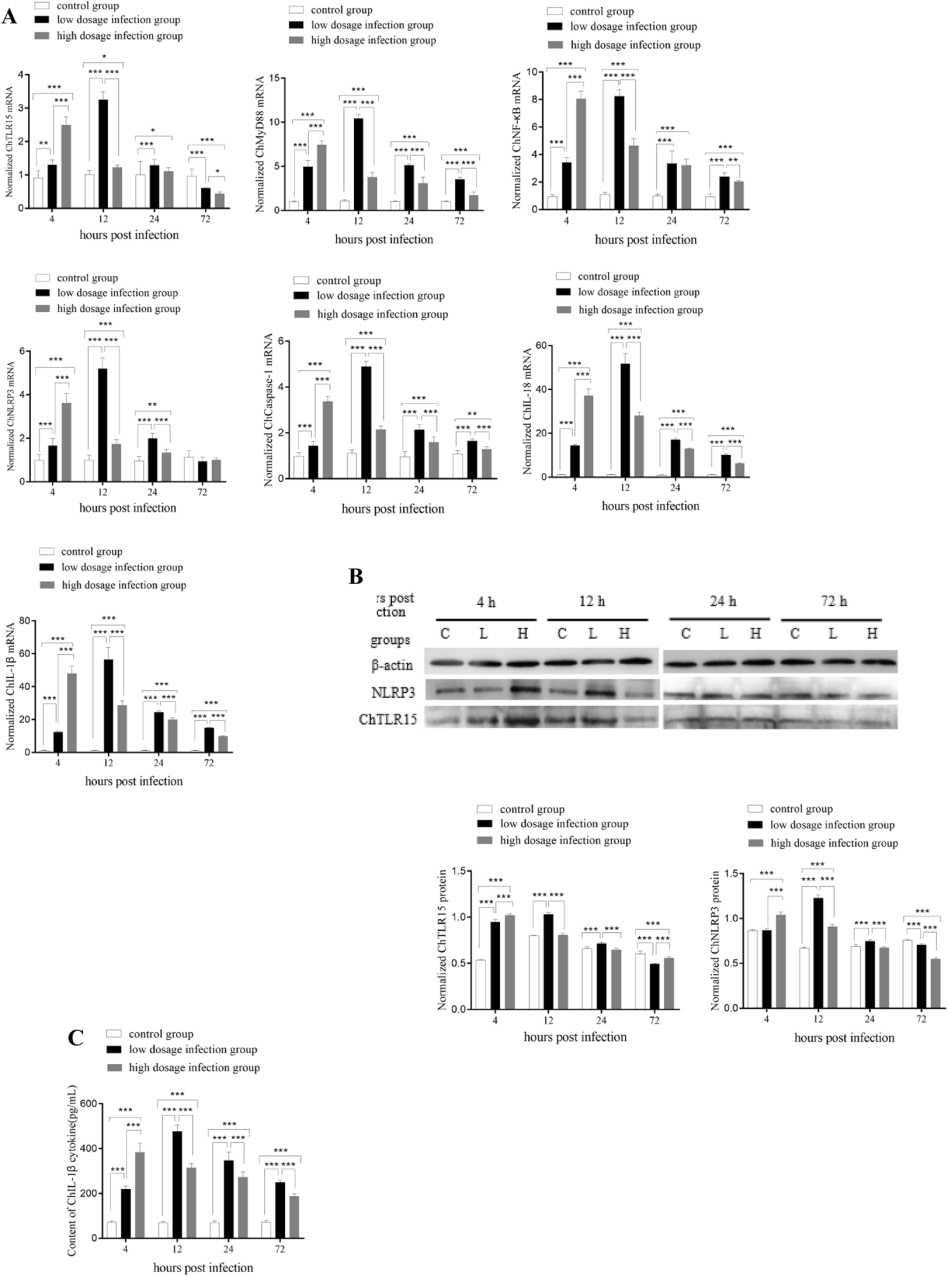
**Expression levels of molecules in the ChTLR15/ChNF-κB-ChNLRP3/ChIL-1β signaling pathway axis in cecal tissues of Eimeria-challenged chickens.** Twenty-one-day-old specific pathogen-free (SPF) chickens were randomly divided into three groups. Each chicken in group 1 (low dosage infection group) was orally challenged with 5000 *E. tenella* sporulated oocysts, group 2 (high dosage infection group) with 30 000 *E. tenella* sporulated oocysts, and group 3 (control group) with 500 μL of PBS (pH 7.2). Ceca from chickens in each group (n = 5) were sampled at 4, 12, 24 and 72 h post-infection. **A** mRNA expression levels of ChTLR15, ChMyD88, Ch ChNF-κB, ChNLRP3, ChCaspase-1, ChIL-18 and ChIL-1β were quantified by quantitative real-time PCR (qPCR). Chicken β-actin was used as a reference gene. **B** Protein expression levels of ChTLR15 and ChNLRP3 were determined by Western blot. Capital letter C represents the control group; L represents the low-dosage infection group; H represents the high-dosage infection group. **C** Protein levels of ChIL-1β in cecal tissues were determined using an ELISA Kit (Jiancheng Bioengineering Institute, Nanjing, China) according to the manufacturer's instructions. *Indicates a significant difference (^*^p < 0.05, ^**^p < 0.01, ^***^p < 0.001).

At 4, 12 and 24 h post-infection (pi) with *E. tenella*, the mRNA expression levels of ChTLR15, ChMyD88, ChNF-κB, ChNLRP3, ChCaspase-1, ChIL-1β and ChIL-18 (Figure 2A) in the ceca of chickens from the two infected groups were all significantly higher than those in the control group (*p* < 0.05). For the high-dosage infection group (challenged with 30 000 sporulated oocysts), the mRNA expression levels of all molecules reached peak levels at 4 h pi and then gradually decreased at 12, 24 and 72 h pi but were still significantly higher than those of the control group (*p* < 0.05). For the low-dosage infection group (challenged with 5000 sporulated oocysts), the mRNA expression levels of all target molecules gradually increased and reached peak levels at 12 h pi and then gradually decreased at 24 and 72 h pi but were still significantly higher than those of the control group (*p* < 0.01). The protein expression levels of ChTLR15 and ChNLRP3 (Figure 2B) and ChIL-1β (Figure 2C) in the ceca of *Eimeria*-infected chickens exhibited similar dynamic changes at 4, 12, 24 and 72 h pi. These results indicated that both the ChTLR15/ChNF-κB and ChNLRP3/ChIL-1β pathways were activated in the sporozoite stage during *E. tenella* infection.

### Expression levels of molecules in the ChTLR15 and ChNLRP3 pathways in DF1 cells

**Figure 3:**
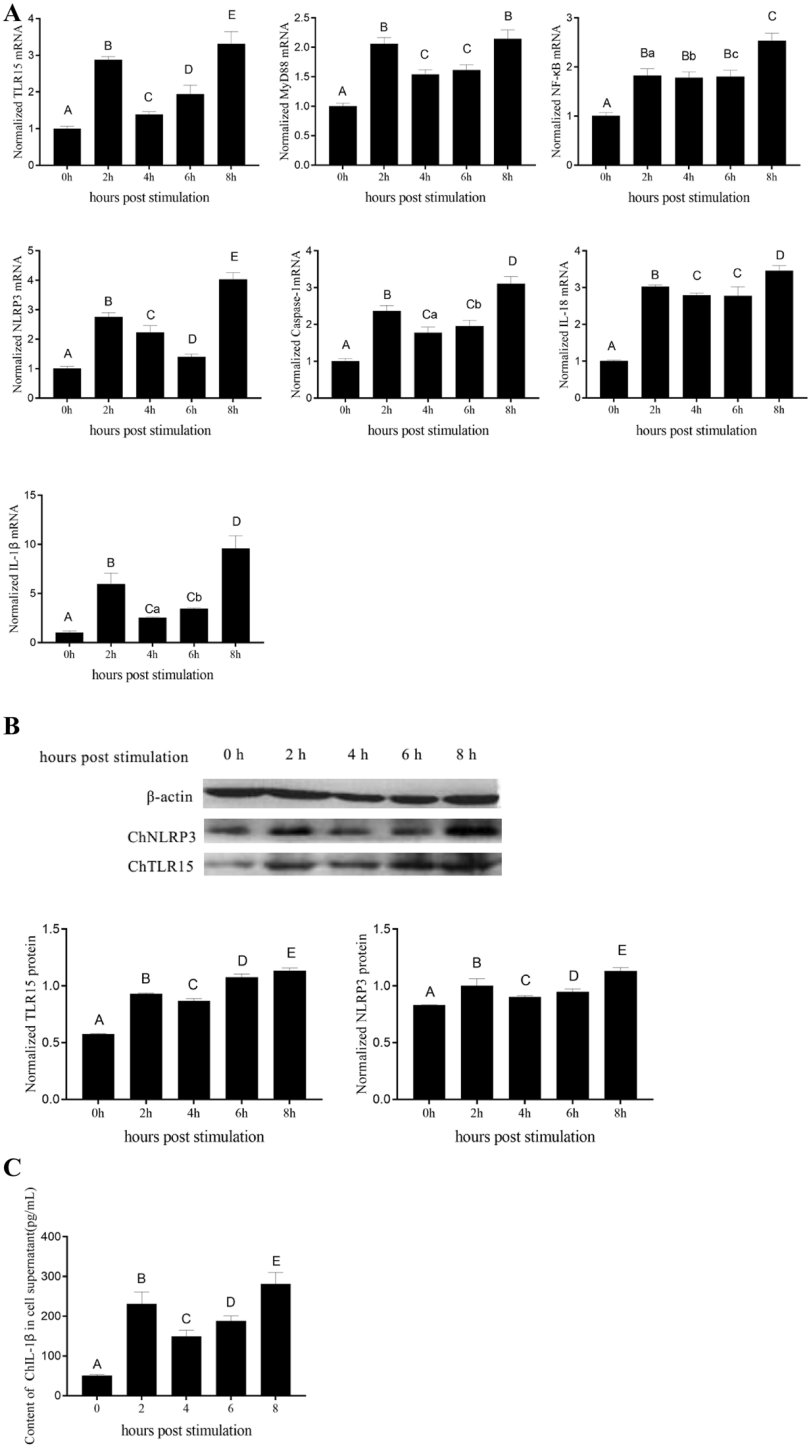
**Expression levels of molecules in the ChTLR15/ChNF-κB-ChNLRP3/ChIL-1β signaling pathway in DF1 cells stimulated by E. tenella sporozoites.** **A** Monolayer DF1 cells were stimulated by purified *E. tenella* sporozoites. At 0, 2, 4, 6, and 8 h post-stimulation, the mRNA levels of ChTLR15, ChMyD88, ChNF-κB, ChNLRP3, ChCaspase-1, ChIL-1β and ChIL-18 in DF1 cells were quantified by quantitative real-time PCR (qPCR). Chicken β-actin was used as a reference gene. **B** Protein levels of ChTLR15 and ChNLRP3 in DF1 cells were detected by Western blot at 0, 2, 4, 6, and 8 h post-stimulation. **C** Protein levels of ChIL-1β in culture supernatant were analyzed using an ELISA kit (Jiancheng Bioengineering Institute, Nanjing, China) at 0, 2, 4, 6, and 8 h post-stimulation. Highly significant differences (*p* < 0.01) between numbers with different capital letters. Significant differences (*p* < 0.05) between numbers with different lowercase letters. No significant difference (*p* > 0.05) between numbers with the same letter.

At 2, 4, 6 and 8 h post-stimulation with sporozoites, the mRNA expression levels of ChTLR15, ChMyD88, ChNF-κB, ChNLRP3, ChCaspase-1, ChIL-1β and ChIL-18 (Figure 3A) and the protein expression levels of ChTLR15 and ChNLRP3 (Figure 3B) in DF1 cells stimulated by sporozoites were significantly higher than those in cells without stimulation (*p* < 0.01). The protein expression levels of ChIL-1β in the supernatants of stimulated cells were also significantly higher than those in cells without stimulation (*p* < 0.01) (Figure 3C). The above results further showed that both the ChTLR15/ChNF-κB and ChNLRP3/ChIL-1β pathways were activated by *E. tenella* sporozoites.

### Interference with ChTLR15 downregulated the expression of molecules in the ChNLRP3 pathway

**Figure 4:**
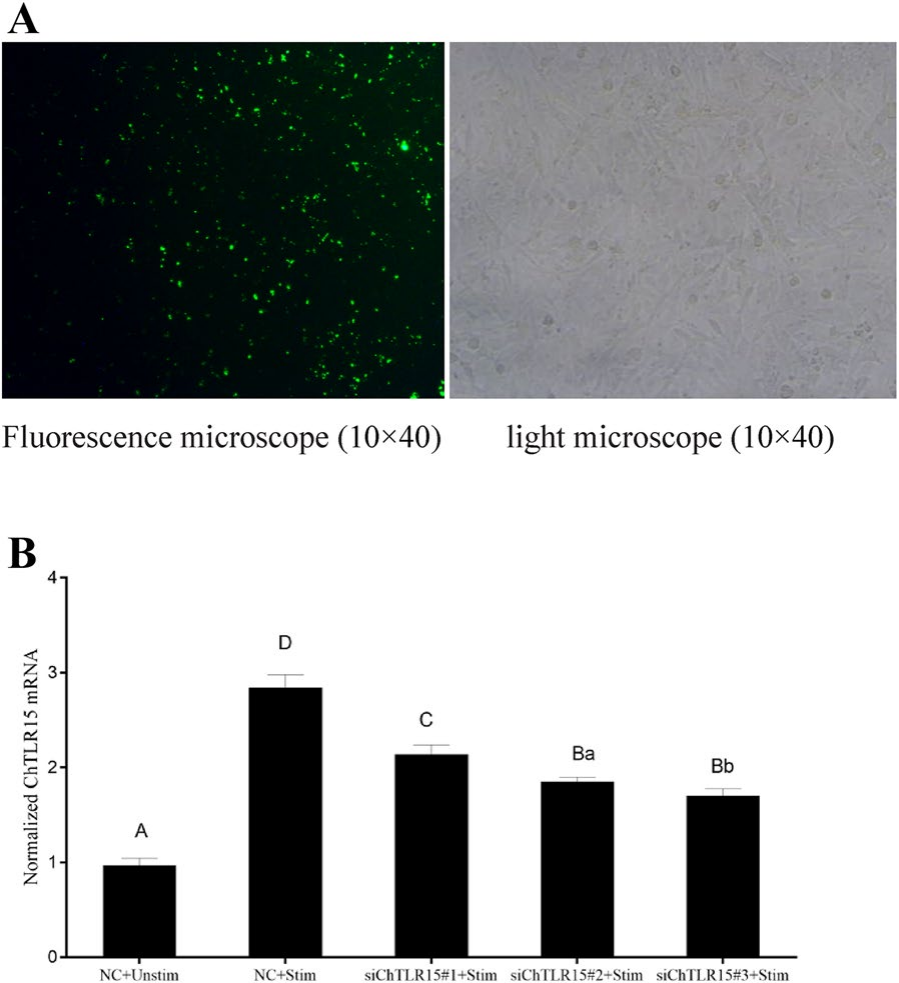
**Transfection efficiency of three target siRNAs for ChTLR15.** **A** To ensure the transfection efficiency of three target siRNAs for ChTLR15, the concentrations of terminal fluorescently labeled siRNA (FAM Negative Control) and Lipofectamine 2000 were optimized according to the manufacturer’s instructions. The final concentration of 50 nM siRNA and 5 μL of Lipofectamine 2000 were chosen. The transfection effects were observed by fluorescence microscopy (left, 10 × 40) and light microscopy (right, 10 × 40). **B** Three ChTLR15 siRNAs (siChTLR15#1, siChTLR15#2 and siChTLR15#3) were transfected into DF1 monolayer cells (1.5 × 10^6^ cells per well) in 6-well plates. At 24 h post-transfection, DF1 cells in each well were stimulated with purified *E. tenella* sporozoites (4 × 10^5^ sporozoites per well) for 2 h. DF1 cells transfected with negative control (NC) siRNA and stimulated with (NC + stim) or without (NC + Unstim) purified sporozoites were designed as controls. The interference efficiency of three ChTLR15 siRNAs was compared by quantifying the mRNA level of ChTLR15. siChTLR15#1 showed the highest interference efficiency and was chosen for subsequent experiments. Highly significant differences (*p* < 0.01) between numbers with different capital letters. Significant differences (*p* < 0.05) between numbers with different lowercase letters. No significant difference (*p* > 0.05) between numbers with the same letter.

**Figure 5:**
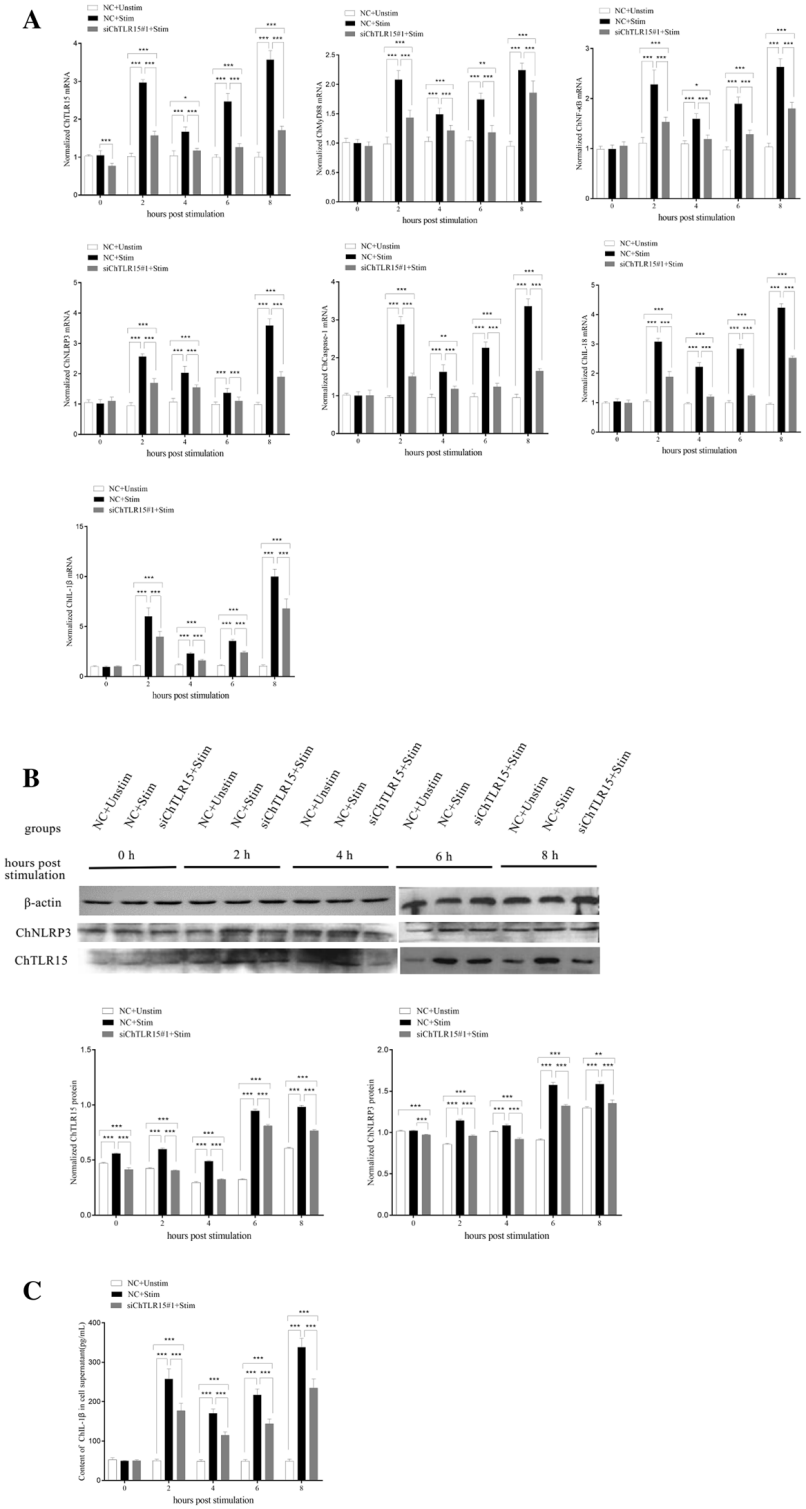
**Interference with ChTLR15 downregulated the expression levels of molecules in the ChTLR15/ChNF-κB-ChNLRP3/ChIL-1β signaling pathway. A** Monolayer DF1 cells (1.5 × 10^6^ cells per well) were treated with siChTLR15#1 for 2 h and then stimulated with purified *E. tenella* sporozoites (4 × 10^5^ sporozoites per well). At 0, 2, 4, 6, and 8 h post-stimulation, cells in each group were collected to quantify the mRNA levels of ChTLR15, ChMyD88, ChNF-κB, ChNLRP3, ChCaspase-1, ChIL-18 and ChIL-1β by quantitative real-time PCR (qPCR). Chicken β-actin was used as a reference gene. **B** DF1 cells transfected with negative control (NC) siRNA and stimulated with (NC + Stim) or without (NC + Unstim) *E. tenella* sporozoites were designed as controls. Protein expression levels of ChTLR15 and ChNLRP3 in cells were determined by Western blot at 0, 2, 4, 6, and 8 h post-stimulation. **C** Protein expression levels of ChIL-1β in culture supernatant were analyzed using an ELISA kit (Jiancheng Bioengineering Institute, Nanjing, China) at 0, 2, 4, 6, and 8 h post-stimulation. ^*^Indicates a significant difference (^*^p < 0.05, ^**^p < 0.01, ^***^p < 0.001).

The RNA interference technique was used to knock down the expression of ChTLR15 in DF1 cells. The results showed that the mRNA expression levels of ChTLR15 in DF1 cells were significantly downregulated at a final concentration of 50 nM siChTLR15#1 and 5 μL of Lipofectamine 2000 (*p* < 0.01) (Figure 4). At 2, 4, 6 and 8 h post-stimulation with sporozoites, the mRNA expression levels of all molecules in the ChTLR15/ChNF-κB and ChNLRP3/ChIL-1β pathways (Figure 5A), protein expression levels of ChTLR15 and ChNLRP3 (Figure 5B) in cells, and ChIL-1β protein levels in the supernatant of ChTLR15-knockdown cells were all highly significantly downregulated compared to cells in the negative control (NC) groups with (NC + Stim) or without (NC + Unstim) stimulation by sporozoites (*p* < 0.001) (Figure 5C). The dynamic changes and tendencies of molecules in the ChTLR15/ChNF-κB pathway were consistent with those in the ChNLRP3/ChIL-1β pathway, which suggested that *E. tenella* sporozoites specifically activated the ChTLR15/ChNF-κB signaling pathway and then further triggered the ChNLRP3/ChIL-1β pathway.

### Overexpressed ChTLR15 upregulated the expression of molecules in the ChNLRP3 pathway

**Figure 6:**
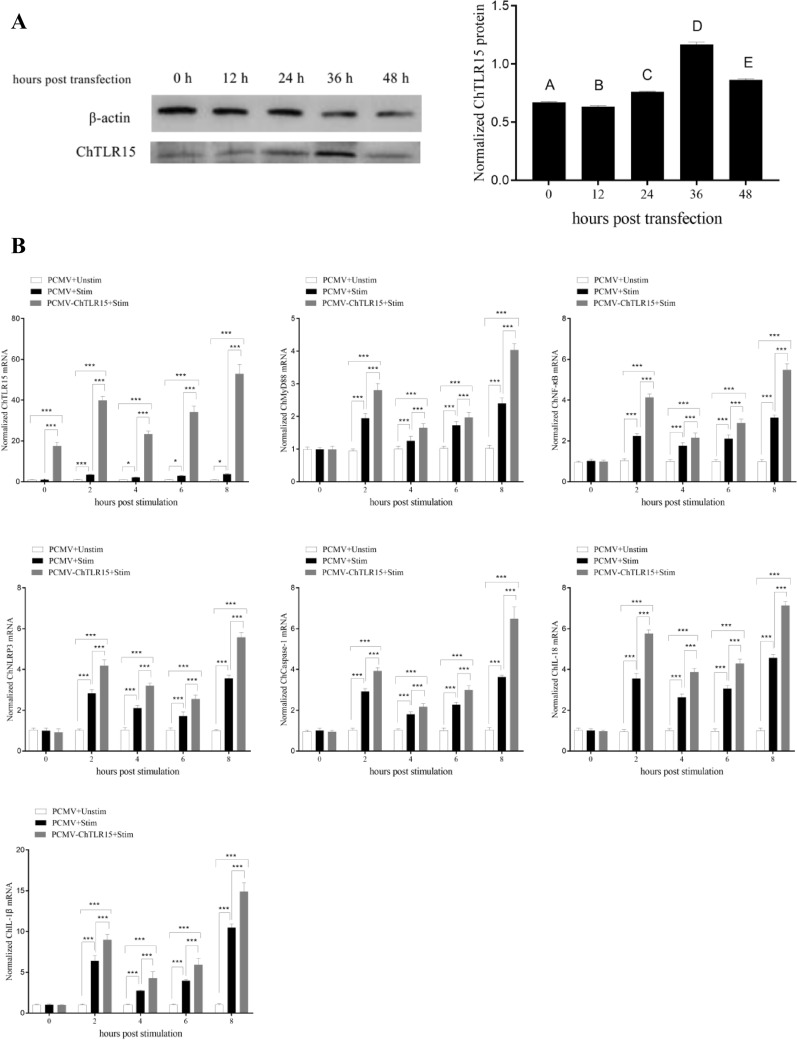

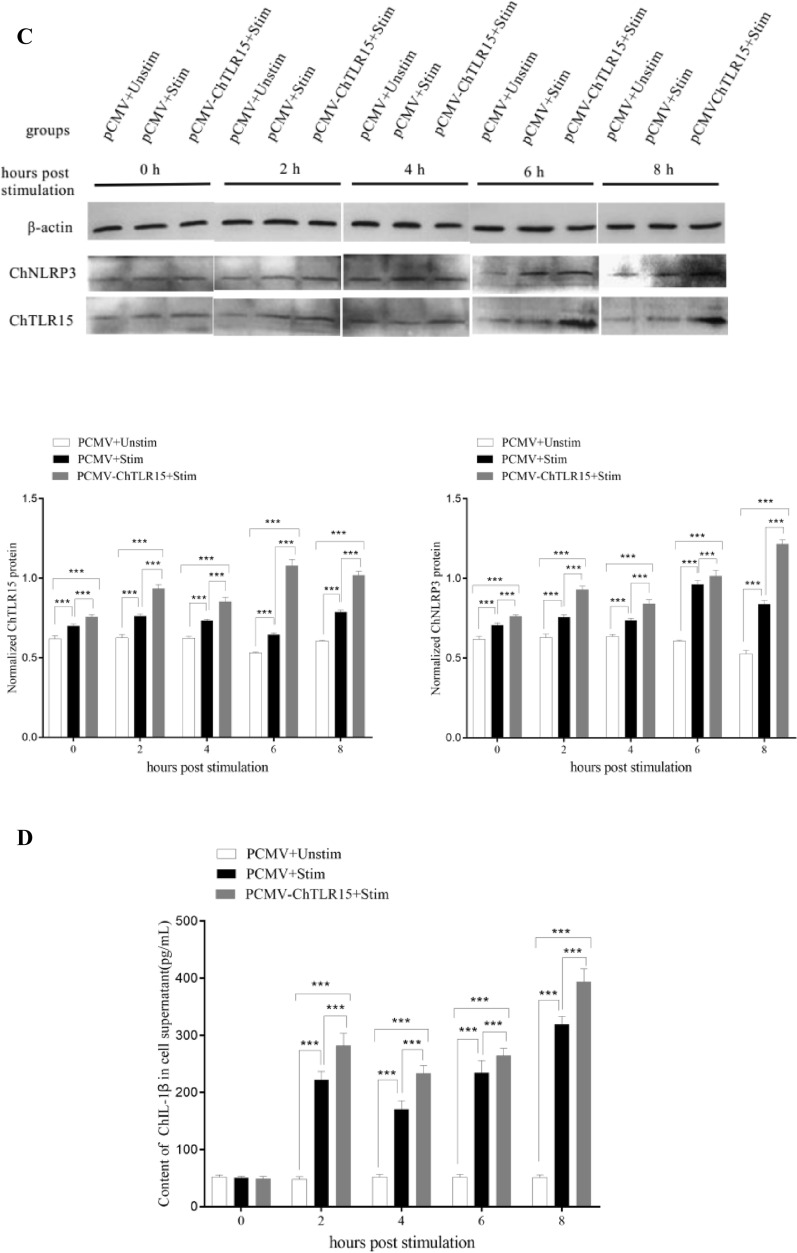
**Overexpressed ChTLR15 upregulated the expression levels of molecules in the ChTLR15/ChNF-κB-ChNLRP3/ChIL-1β signaling pathway.** **A** pCMV-ChTLR15 plasmid was transfected into DF1 monolayer cells cultured in 6-well plates using Lipofectamine 2000 diluted according to the manufacturer’s protocol. ChTLR15 protein levels in transfected cells were highly significantly upregulated (*p* < 0.01) at 36 h post-transfection compared to other time points. **B** At 36 h post-transfection with pCMV-ChTLR15, DF1 cells in each group were stimulated by purified *E. tenella* sporozoites (4 × 10^5^ sporozoites per well). Cells transfected with empty plasmid pCMV and stimulated with (pCMV + Stim) or without (pCMV + Unstim) sporozoites were used as controls. At 0, 2, 4, 6, and 8 h post-stimulation, cells in each group were collected to quantify the mRNA levels of ChTLR15, ChMyD88, ChNF-κB, ChNLRP3, ChCaspase-1, ChIL-18 and ChIL-1β by quantitative real-time PCR (qPCR). **C** Protein expression levels of ChTLR15 and ChNLRP3 in DF1 cells were detected by Western blot at 0, 2, 4, 6, and 8 h post-stimulation. **D** Protein expression levels of ChIL-1β in culture supernatant were analyzed using an ELISA kit (Jiancheng Bioengineering Institute, Nanjing, China) at 0, 2, 4, 6, and 8 h post-stimulation.

At 36 h post-transfection with pCMV-ChTLR15, the expression of ChTLR15 protein in DF1 cells was highly significantly upregulated (*p* < 0.01) (Figure 6A). At 0, 2, 4, 6 and 8 h post-stimulation with sporozoites, the mRNA expression levels of all molecules in the ChTLR15/ChNF-κB and ChNLRP3/ChIL-1β pathways (Figure 6B), the protein expression levels of ChTLR15 and ChNLRP3 (Figure 6C) in cells, and ChIL-1β protein levels in the supernatant of cells transfected with pCMV-ChTLR15 were all significantly higher than those in pCMV-transfected cells with (pCMV + Stim) or without (pCMV + Unstim) stimulation by sporozoites (*p* < 0.001) (Figure 6D). All molecules contained in the ChTLR15/ChNF-κB and ChNLRP3/ChIL-1β pathways showed consistent varying tendencies, which further clarified that *E. tenella* sporozoites specifically activated the ChTLR15/ChNF-κB signaling pathway and then triggered activation of the ChNLRP3/ChIL-1β pathway to mediate inflammatory responses during *Eimeria* infection.

## Discussion

In recent years, researchers have realized that exploration of inhibitors of important signaling pathways involved in the stages of parasite invasion into host cells may be a promising way to develop novel anticoccidial drugs. Toll-like receptors (TLRs) are protein molecules that are closely related to innate immune responses. While we focused on the roles of TLRs during *Eimeria* infection, we are interested in ChTLR15. To date, only a few articles have been published that quantify ChTLR15 mRNA expression levels during infection by parasites [6, 7, 25], bacteria [8, 26] and viruses [27], all of which indicate that ChTLR15 plays a vital role in immune responses against infections. However, the agonist of ChTLR15 is still unknown. In the present study, the results from animal experiments revealed that at 4, 12 and 24 h pi with *E. tenella*, the mRNA expression levels of ChTLR15, ChMyD88, ChNF-κB, ChNLRP3, ChCaspase-1, ChIL-1β and ChIL-18 in cecal tissues of chickens from the two infected groups were significantly higher than those in the control group. The above results indicated that the ChTLR15/ChNF-κB and ChNLRP3/ChIL-1β signaling pathways were involved during *E. tenella* infection. It is widely accepted that sporozoites are released from sporulated oocysts after 1 to 2 days of development in the chicken intestinal tract, which further suggests that *Eimeria* sporozoites are strictly related to the activation of the ChTLR15/ChNF-κB and ChNLRP3/ChIL-1β signaling pathways.

It was reported that the NLRP3 inflammasome plays a crucial role in regulating the activation of inflammatory responses to many pathogenic bacteria. During innate immune responses, NLRP3 recruits Caspase-1 to form inflammatory corpuscles, and then activated Caspase-1 cleaves inactive pro-IL-1β and pro-IL-18 to become active IL-1β and IL-18 and therefore induces inflammatory responses [28]. To date, the roles of ChNLRP3 in the immune responses to *E. tenella* infection have not been reported. In the present study, considering that the expression levels of key molecules involved in both the ChTLR15/ChNF-κB and ChNLRP3/ChIL-1β pathways were highly significantly upregulated in vivo and in vitro, we postulated that *E. tenella* sporozoites specifically activated the ChTLR15/ChNF-κB pathway and then triggered activation of the ChNLRP3/ChIL-1β pathway, and the ChTLR15/ChNF-κB-ChNLRP3/ChIL-1β signaling pathway axis contributed to the inflammatory responses to *Eimeria* infection. Chicken embryo fibroblast (DF-1) cells have been extensively used to study the invasion of *Eimeria* sporozoites and innate immune responses. Therefore, DF1 cells were used to elucidate the sporozoite-specific activation of the ChTLR15/ChNF-κB pathway and further clarify the positive relationship between ChTLR15/ChNF-κB and the ChNLRP3/ChIL-1β pathway. RNAi and overexpression technology were employed to downregulate and upregulate the expression of ChTLR15 protein, respectively. The quantification of key molecules contained in the ChTLR15/ChNF-κB and ChNLRP3/ChIL-1β pathways in DF1 cells was consistent with our expectations. In experiments investigating the knockdown and overexpression of ChTLR15, the dynamic change tendencies of all molecules in the ChNLRP3/ChIL-1β pathway corresponded with those in the ChTLR15/ChNF-κB pathway, indicating that changes in ChTLR15 effectively regulated the expression of key molecules in the ChNLRP3/ChIL-1β pathway.

NLRP3 is an endogenous molecule, and the processes of transcription, translation, activation and assembly are carried out in the cytoplasm of cells [29]. Therefore, *Eimeria* sporozoites cannot directly stimulate the activation of ChNLRP3 during invasion. It is possible that the assembly of the ChNLRP3 inflammasome is initiated by upstream molecules involved in certain signaling pathways. The signal transduction pathways that modulate inflammasomes are widely recorded, among which NF-κB is commonly accepted as responsible for regulating the activation of the NLRP3 inflammasome and IL-1β production [30, 31]. NF-κB is widely distributed in cells and participates in the regulation of the expression of various genes, including inflammatory and immune responses [30]. The in vitro results clearly indicated that *E. tenella* sporozoites specifically activated the ChTLR15/ChNF-κB pathway and then triggered activation of the ChNLRP3/ChIL-1β pathway via crosstalk between ChNF-κB and the ChNLRP3 pathway, which partially mediated inflammatory injury during *E. tenella* infection.

It was previously reported that monocyte-derived macrophages stimulated by heat-killed *E. tenella* sporozoites displayed higher mRNA expression levels of ChTLR15 than live sporozoites [7], which demonstrated that certain heat-stable structural components in sporozoites may be recognized by ChTLR15. Invasion of host cells by sporozoites is a prerequisite for the subsequent developmental stages of *Eimeria* parasites in host epithelial cells. In our subsequent research, the heat-stable structural component in sporozoites was further explored. The present study provides a new idea for preparing anticoccidial drugs based on the identification of inhibitors of the ChTLR15/ChNF-κB-ChNLRP3/ChIL-1β signaling pathway.

## Data Availability

Not applicable.

## Abbreviations

*E. tenella*: *Eimeria tenella*
TLRs: Toll-like receptors
PRRs: Pattern recognition receptors
PAMPs: Pathogen-associated molecular patterns
ChTLR15: Chicken Toll-like receptor 15
ChMyD88: Chicken myeloid differentiation primary response 88
ChNF-*κ*B: Chicken nuclear transcription factor-κB
ChNLRP3: Chicken NOD-like receptor 3
ChCaspase-1: Chicken cysteinyl aspartate specific proteinase 1
ChIL-18: Chicken interleukin 18
ChIL-1β: Chicken interleukin 1 Beta
DF-1: Chicken embryo fibroblast cells
SPF: Specific pathogen-free
ELISA: Enzyme-linked immunosorbent assay
HRP: Horseradish peroxidase
OD600: Optical density at 600 nm
IPTG: Isopropyl-b-d-thiogalactopyranoside
SDS-PAGE: Sodium dodecyl sulfate-polyacrylamide gel electrophoresis
siRNA: Small interfering RNA
NC: Negative control
qPCR: Quantitative real-time PCR
MIQE: Minimum information for publication of quantitative real-time PCR experiments
ANOVA: One-way analysis of variance
SD: Standard deviation
pi: Post-infection

## References

[1] Dalloul RA, Lillehoj HS (2006). Poultry coccidiosis: recent advancements in control measures and vaccine development. Expert Rev Vaccines.

[2] Blake DP, Tomley FM (2014). Securing poultry production from the ever present *Eimeria* challenge. Trends Parasitol.

[3] Shirley MW, Smith AL, Blake DP (2007). Challenges in the successful control of the avian coccidia. Vaccine.

[4] Chapman HD (2014). Milestones in avian coccidiosis research: a review. Poult Sci.

[5] Zhao Z, Zhao Q, Zhu S, Huang B, Lv L, Chen T, Yan M, Han H, Dong H (2019). iTRAQ-based comparative proteomic analysis of cells infected with *Eimeria tenella* sporozoites. Parasite.

[6] Zhang L, Liu R, Ma L, Wang Y, Pan B, Cai J, Wang M (2012). *Eimeria tenella*: expression profiling of toll-like receptors and associated cytokines in the cecum of infected day-old and three-week old SPF chickens. Exp Parasitol.

[7] Zhou Z, Wang Z, Cao L, Hu S, Zhang Z, Qin B, Guo Z, Nie K (2013). Upregulation of chicken TLR4, TLR15 and MyD88 in heterophils and monocyte-derived macrophages stimulated with *Eimeria tenella* in vitro. Exp Parasitol.

[8] Kogut MH, Chiang HI, Swaggerty CL, Pevzner IY, Zhou H (2012). Gene expression analysis of Toll-Like receptor pathways in heterophils from genetic chicken lines that differ in their susceptibility to *salmonella enteritidis*. Front Genet.

[9] de Zoete MR, Bouwman LI, Keestra AM, van Putten JP (2011). Cleavage and activation of a Toll-like receptor by microbial proteases. Proc Natl Acad Sci U S A.

[10] Guo H, Callaway JB, Ting JP (2015). Inflammasomes: Mechanism of action, role in disease, and therapeutics. Nat Med.

[11] Lima-Junior DS, Costa DL, Carregaro V, Cunha LD, Silva AL, Mineo TW, Gutierrez FR, Bellio M, Bortoluci KR, Flavell RA, Bozza MT, Silva JS, Zamboni DS (2013). Inflammasome-derived IL-1β production induces nitric oxide-mediated resistance to *Leishmania*. Nat Med.

[12] Silva GK, Costa RS, Silveira TN, Caetano BC, Horta CV, Gutierrez FR, Guedes PM, Andrade WA, De Niz M, Gazzinelli RT, Zamboni DS, Silva JS (2013). Apoptosis-associated speck-like protein containing a caspase recruitment domain inflammasomes mediate IL-1beta response and host resistance to *Trypanosoma cruzi* infection. J Immunol.

[13] Gorfu G, Cirelli KM, Melo MB, Mayer-Barber K, Crown D, Koller BH, Masters S, Sher A, Leppla SH, Moayeri M, Saeij JP, Grigg ME (2014). Dual role for inflammasome sensors NLRP1 and NLRP3 in murine resistance to *Toxoplasmagondii*. MBio.

[14] Wang X, Gong P, Zhang N, Li L, Chen S, Jia L, Liu X, Li J, Zhang X (2019). Inflammasome activation restrains the intracellular *Neospora caninum* proliferation in bovine macrophages. Vet Parasitol.

[15] Chen T, Huang B, Zhao Q, Dong H, Zhu S, Zhao Z, Lv L, Yan M, Han H (2018). Molecular characterization and functional analysis of *Eimeria tenella* malate dehydrogenase. Parasitol Res.

[16] Wang L, Zhu S, Zhao Q, Huang B, Lv L, Liu G, Li Z, Zhao H, Han H, Dong H (2019). Effects of host fatty acid-binding protein 4 on *Eimeria tenella* sporozoites invasion of cells. Parasitol Res.

[17] Zhao Y, Zou M, Sun Y, Zhang K, Peng X (2019). gga-miR-21 modulates *Mycoplasma gallisepticum* (HS strain)-Induced inflammation via targeting MAP3K1 and activating MAPKs and NF-κB pathways. Vet Microbiol.

[18] Ma D, Huang Y, Ma C, Zhang L, Wang J, Wang D, Li J, Dalloul RA (2019). *Eimeria tenella*: specific EtAMA1-binding peptides inhibit sporozoite entry into host cells. Poult Sci.

[19] Ma D, Ma C, Pan L, Li G, Yang J, Hong J, Cai H, Ren X (2011). Vaccina-tion of chickens with DNA vaccine encoding *Eimeria acervulina* 3–1Eand chicken IL-15 offers protection against homologous challenge. Exp Parasitol.

[20] Ma C, Zhang L, Gao M, Ma D (2017). Construction of *Lactococcus lactis* expressing secreted and anchored *Eimeria tenella* 3–1E protein and comparison of protective immunity against homologous challenge. Exp Parasitol.

[21] Wang D, Zhang Y, Ma C, Ma D, Zhao Q, Wang F, Huang Y, Li J, Zhang L, Zhou EM (2018). Live recombinant *Lactococcus lactis* expressing avian hepatitis virus ORF2 protein: Immunoprotection against homologous virus challenge in chickens. Vaccine.

[22] Bustin SA, Benes V, Garson JA, Hellemans J, Huggett J, Kubista M, Mueller R, Nolan T, Pfaffl MW, Shipley GL, Vandesompele J, Wittwer CT (2009). The MIQE guidelines: minimum information for publication of quantitative real-time PCR experiments. Clin Chem.

[23] Livak KJ, Schmittgen TD (2001). Analysis of relative gene expression data using real-time quantitative PCR and the 2(-Delta Delta C(T)) Method. Methods.

[24] Motulsky HJ (2003) Prism 4 statistics guide-statistical analyses for laboratory and clinical researchers. GraphPad Software lnc., San Diego CA, www.graphpad.com.

[25] Sumners LH, Miska KB, Jenkins MC, Fetterer RH, Cox CM, Kim S, Dalloul RA (2011). Expression of Toll-like receptors and antimicrobial peptides during *Eimeria praecox* infection in chickens. Exp Parasitol.

[26] Higgs R, Cormican P, Cahalane S, Allan B, Lloyd AT, Meade K, James T, Lynn DJ, Babiuk LA, O’Farrelly C (2006). Induction of a novel chicken toll-like receptor following *salmonella enterica serovar typhimurium* infection. Infect Immun.

[27] Jie H, Lian L, Qu L, Zheng J, Hou Z, Xu G, Song J, Yang N (2013). Differential expression of Toll-like receptor genes in lymphoid tissues between Marek's disease virus-infected and noninfected chickens. Poult Sci.

[28] Atianand MK, Rathinam VA, Fitzgerald KA (2013). SnapShot: inflammasomes. Cell.

[29] Sutterwala FS, Haasken S, Cassel SL (2014). Mechanism of NLRP3 inflammasome activation. Ann N Y Acad Sci.

[30] He Y, Hara H, Núñez G (2016). Mechanism and regulation of NLRP3 inflammasome activation. Trends Biochem Sci.

[31] Greten FR, Arkan MC, Bollrath J, Hsu LC, Goode J, Miething C, Goktuna SI, Neuenhahn M, Fierer J, Paxian S, Van Rooijen N, Xu Y, O'Cain T, Jaffee BB, Busch DH, Duyster J, Schmid RM, Eckmann L, Karin M (2007). NF-kappaB is a negative regulator of IL-1beta secretion as revealed by genetic and pharmacological inhibition of IKKbeta. Cell.

